# MRMPlus: an open source quality control and assessment tool for SRM/MRM assay development

**DOI:** 10.1186/s12859-015-0838-z

**Published:** 2015-12-12

**Authors:** Paul Aiyetan, Stefani N. Thomas, Zhen Zhang, Hui Zhang

**Affiliations:** Department of Pathology, The Johns Hopkins University School of Medicine, Robert H and Clarice Smith Building, Room 4000C, 400 North Broadway, Baltimore, MD 21287 USA

## Abstract

**Background:**

Selected and multiple reaction monitoring involves monitoring a multiplexed assay of proteotypic peptides and associated transitions in mass spectrometry runs. To describe peptide and associated transitions as stable, quantifiable, and reproducible representatives of proteins of interest, experimental and analytical validation is required. However, inadequate and disparate analytical tools and validation methods predispose assay performance measures to errors and inconsistencies.

**Results:**

Implemented as a freely available, open-source tool in the platform independent Java programing language, MRMPlus computes analytical measures as recommended recently by the Clinical Proteomics Tumor Analysis Consortium Assay Development Working Group for “Tier 2” assays – that is, non-clinical assays sufficient enough to measure changes due to both biological and experimental perturbations. Computed measures include; limit of detection, lower limit of quantification, linearity, carry-over, partial validation of specificity, and upper limit of quantification.

**Conclusions:**

MRMPlus streamlines assay development analytical workflow and therefore minimizes error predisposition. MRMPlus may also be used for performance estimation for targeted assays not described by the Assay Development Working Group. MRMPlus’ source codes and compiled binaries can be freely downloaded from https://bitbucket.org/paiyetan/mrmplusgui and https://bitbucket.org/paiyetan/mrmplusgui/downloads respectively.

**Electronic supplementary material:**

The online version of this article (doi:10.1186/s12859-015-0838-z) contains supplementary material, which is available to authorized users.

## Background

Selected or Multiple reaction monitoring (SRM and MRM) mass spectrometry is the most widely used MS-based targeted proteomic approach. In contrast to discovery proteomics [1–6], targeted proteomics strategies entail limiting the number of features monitored and optimizes chromatography, instrument tuning, and acquisition methods to achieve high sensitivity [7]. Its advantages over traditional low-throughput quantitative approaches such as ELISA, Western blotting, and immunohistochemistry include multiplexing, a precise relative and absolute quantification of endogenous analytes, no antibody requirement, and an ability to detect unmodified and posttranslationally modified forms of proteins [8–13]. These limitations and opportunities presented by SRM/MRM are well articulated by Shi et al. [14]. As surrogates to protein expression level quantification methods such as mRNA microarray and RNASeq methods fall short of an ability to detect posttranslationally modified proteins, the demand for SRM or MRM-based analytical approaches is anticipated to increase [7].

Similar to parallel reaction monitoring (PRM), MRM typically involves monitoring a multiplexed assay of proteotypic peptides. However under conditions that permit high resolution and high mass accuracy, only a set of preselected transitions are monitored in MRM as opposed to all transitions in PRM [15, 16]. An MRM assay development workflow may be broadly sub-divided into a pre-mass spectrometry acquisition phase and a post-acquisition phase. The pre-acquisition phase includes, 1) generation of target protein list, 2) selection of proteotypic peptides and 3) an experimental design step [17]. The post-acquisition phase recently described by Colangelo et al [7] entails four major steps; 1) peak detection, integration and quantification, 2) data quality assessment, 3) data visualization and exploratory analysis, and 4) fold change/statistical significance analysis.

Irrespective of the targeted proteomics approach employed, the complexities of media within which monitored proteotypic peptides that represent proteins of interest reside result in the somewhat unpredictable analytical behavior of peptides and transitions. Thus, it is important that experimental and analytical validation is performed to describe peptides and associated transitions as stable, quantifiable, and reproducible representatives of proteins of interest.

To standardize and validate quantifiable targeted mass spectrometry-based peptide and protein quantifications, the National Cancer Institute (NCI) National Institutes of Health through the Clinical Proteomic Tumor Analysis Consortium (CPTAC) Assay Development Working Group (ADWG) recently described guidelines, experiments and analytical measures for MRM assay characterization [18]. Extending previously published guidelines and studies [19, 20], the described guidelines ensure some levels of confidence for assayed peptides and proteins in quantitative targeted proteomics studies.

**Fig. 1:**
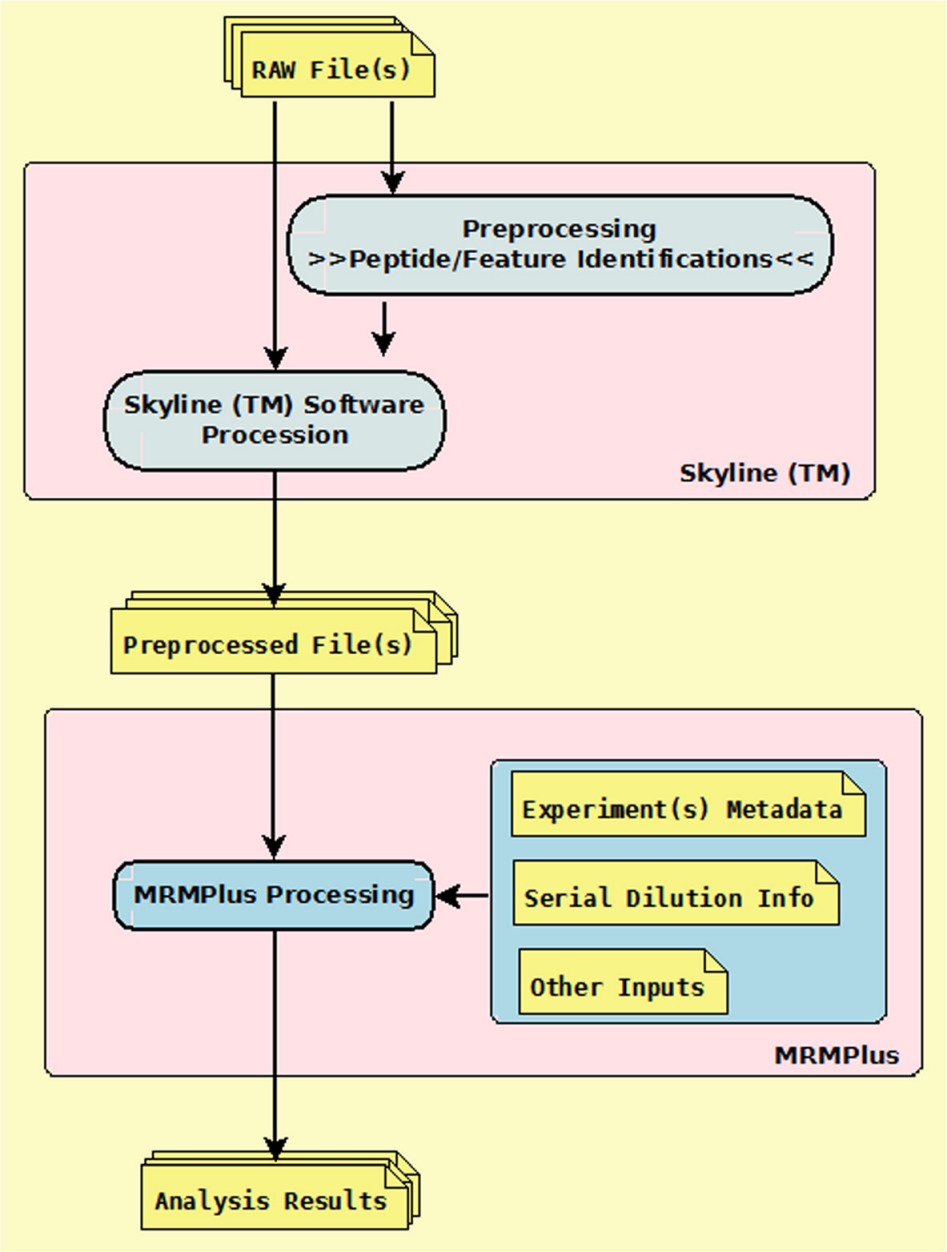
MRMPlus Concept/Flow Diagram. Mass spectrometry data are first preprocessed in Skyline™ and subsequently fed as input to MRMPlus. In addition, MRMPlus takes as inputs, user-defined experiment metadata and a serial dilution information file

Currently available commercial, proprietary tools and open source, vendor-neutral analytical tools for post-data acquisition processing are extensively described in Cham et al [21], Brusniak et al [17], Colangelo et al [7] and Mani et al [19]. Though these tools are highly credible in addressing individual analytical challenges, we found no suitable standalone, platform-independent tool that readily implements the CPTAC ADWG’s recommendation for the evaluation of assay performance. However, Skyline [22], described as the most complete open source platform addressing a great deal of the analytical requirements of an MRM assay development protocol, provided a sufficient basis for the development of MRMPlus. Streamlining workflow (Fig. 1) and minimizing error predisposition, MRMPlus takes as input, Skyline derived preprocessed files (Additional file 1: Table S1), in addition to user defined metadata files (Additional files 2 and 3: Tables S2 and S3) to compute analytical and validation measures as recommended and described in the CPTAC ADWG published guidelines (https://assays.cancer.gov/guidance-document/).

Although MRMPlus was conceptualized and implemented to compute the performance of assays developed according to guidelines established by the CPTAC Assay Development Working Group, it may also be used for performance calculations for targeted assays not described by the Working Group.

## Implementation

**Fig. 2:**
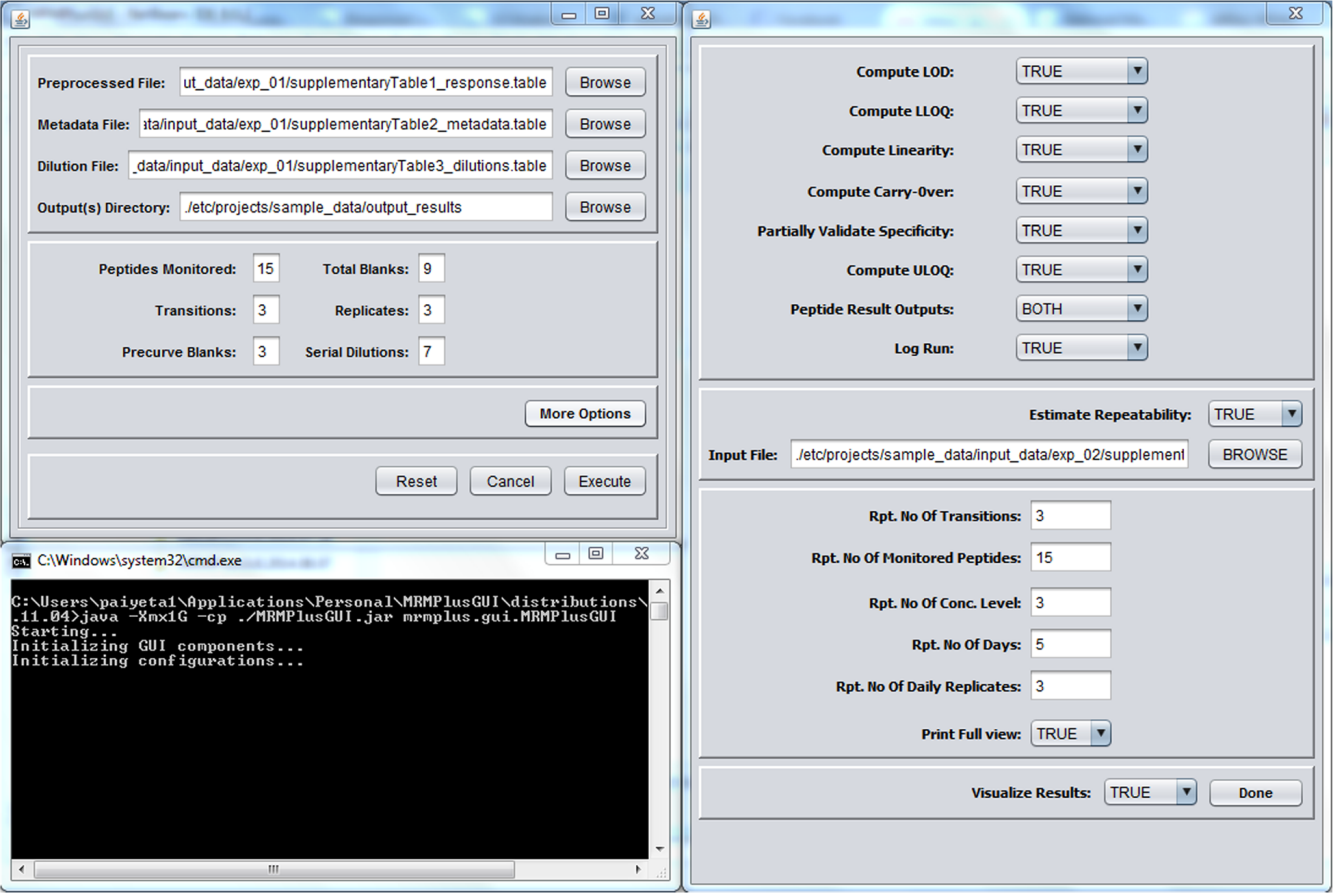
MRMPlus’ Graphics User Interface. In addition to user-defined and Skyline input files, MRMPlus’ interface requests other program configuration options

We implemented MRMPlus in the platform-independent Java programing language (Fig. 2). For statistical evaluations, we utilized classes defined in the Apache Commons Mathematics library [23]. For visualizations, we utilized Java classes defined in the JFreeChart library [http://www.jfree.org/jfreechart/].

Inputs to the program include Skyline-derived output files in tab-delimited format (Additional files 1 and 4: Tables S1 and S4), a user defined experiment metadata (Additional file 2: Table S2), and a dilution information data file (Additional file 3: Table S3). Current implementation of MRMPlus computes analytical measures for “Response Curve” and “Mini-Validation of Repeatability” experiments according to the ADWG’s current specifications (Additional files 5 and 6: Tables S5 and S6). The response curve serves to identify the lower limit of quantification (LLOQ), the limit of detection (LOD), and the linear range of a peptide of interest in a multipoint serial dilution of a biological replicate or matrix. The mini-validation of repeatability experiments attempt to replicate peptide measurements within a complex mixture over multiple days. Its derived metrics approximate the variability of the assay in real-life practice.

Default runtime initiated interface to MRMPlus accepts inputs to compute “Response Curve” analytical measures and the ‘More Options’ button instantiates the “Mini-Validation of Repeatability” module (Fig. 2). Users may modify the configuration file (MRMPlus.config) in the application package to set runtime default options.

Setting the ‘Visualize Results’ option on the ‘More Options’ panel to ‘TRUE’ instructs MRMPlus to generate performance associated visualizations (Fig. 2). MRMPlus generates 8 visualization panels to provide a global perspective of assay performance across all assayed peptide transitions. In addition, MRMPlus provides peptide-specific (‘Response Curve’ and ‘Repeatability’) performance visualizations. (Additional files 7: Figure S1 and Additional file 8: Figure S2).

### Ethics approval

The experimental work that was conducted in this study did not require ethics approval. No patient-derived specimens were used to generate the data for the development of MRMPlus. All of the data were derived from formerly N-linked glycopeptides enriched from commercially-available serum and spiked with crude peptides.

## Results and discussions

To demonstrate MRMPlus’ capabilities, we employed it to compute the assay performance metrics on our panel of formerly N-linked glycopeptides. Peptides were selected from in-house discovery phase proteomic experiment analyses of prostate cancer tissues and serum studies. MRM assays to these were developed using commercially available serum and heavy labeled synthetic peptides as spiked-ins. (Please see https://assays.cancer.gov/guidance-document/ for detailed description of experiments). Here in this case study, we subset fifteen of the profiled peptides and their respectively associated transitions for MRMPlus’ demonstration purpose.

**Fig. 3:**
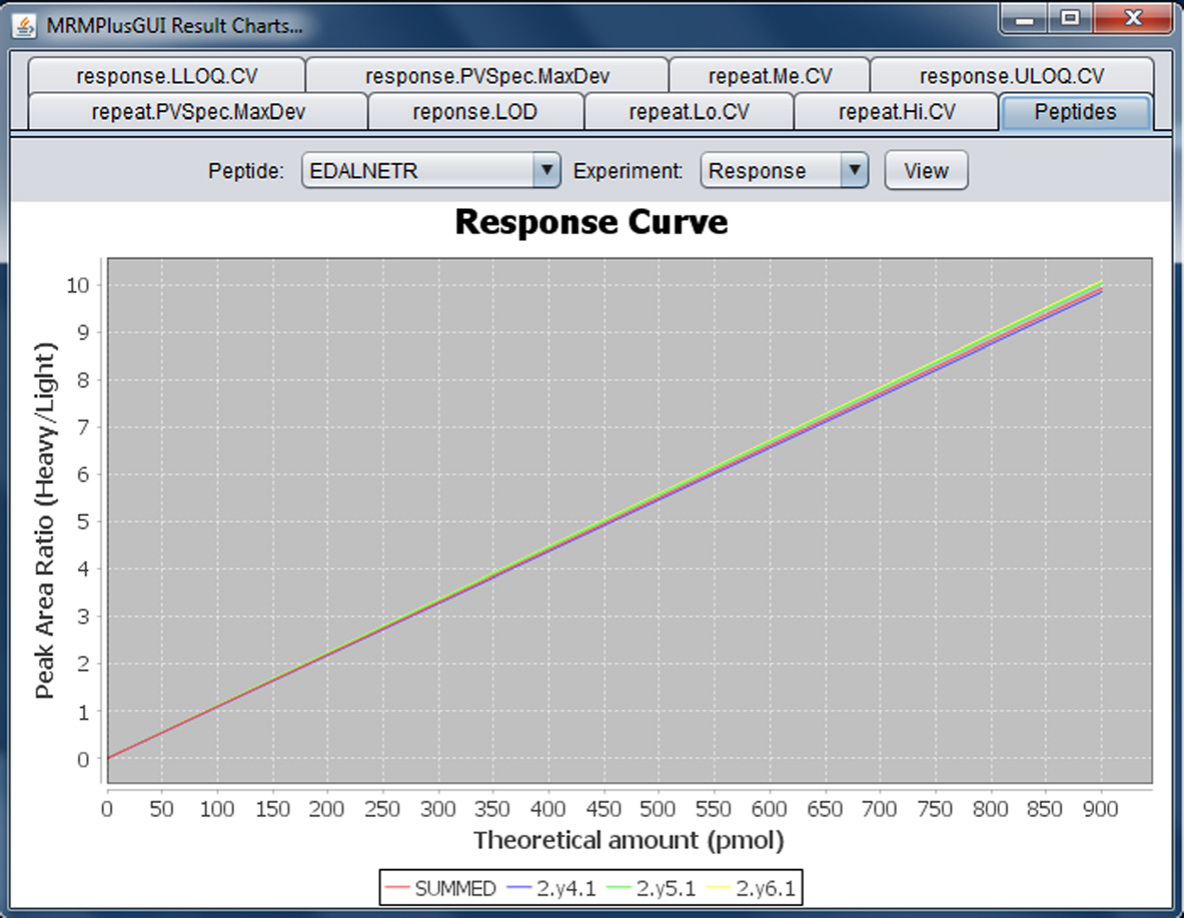
MRMPlus‘ Visualization – Response Curve. For each monitored transition of a particular peptide, experimental values are derived at serial dilution levels (calibration points). Experiments at respective dilution level are performed in replicates. Figure shows MRMPlus-generated response curves for a sample peptide – EDALNETR. It includes performance plots for the peptide associated transitions (2.y4.1, 2.y5.1, and 2.y6.1) and summed transition

**Fig. 4:**
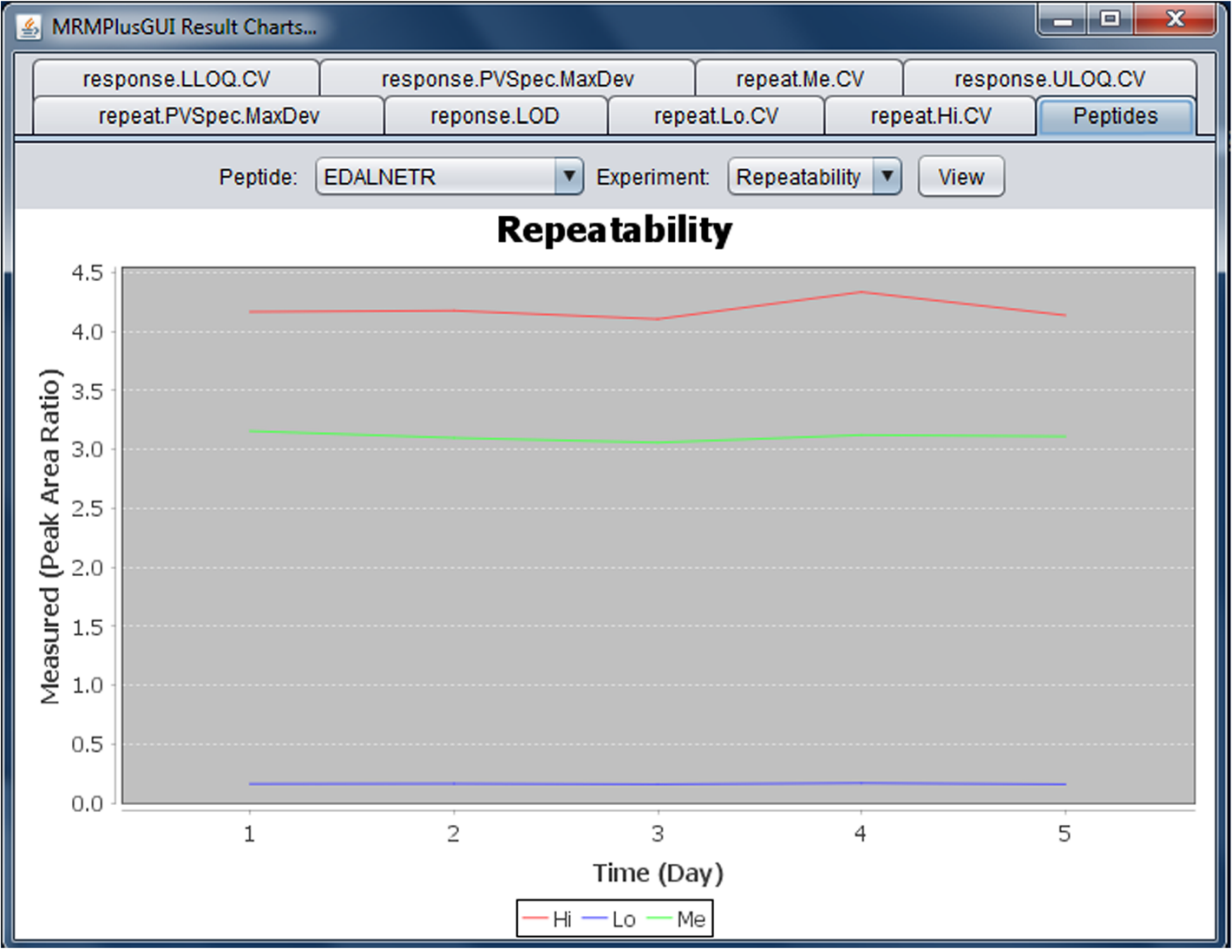
MRMPlus‘ Visualization – Mini-validation of Repeatability. For each monitored transition, low, medium and high concentration assays are profiled over five days with replicate experiments on each day. Figure shows MRMPlus-generated ‘repeatability’ performance for a sample peptide – EDALNETR. It includes performance plots for the summed transition at the a low, medium and high concentration level. Y-axis coordinates are median estimate of replicate measurements at the different concentration levels while the X-axis coordinates are the days when the experiments were performed. Current implementation of MRMPlus [by default] generates repeatability performance plot for peptides summed transition

The current implementation of MRMPlus outputs results for ADWG-defined experiments’ ‘Response Curve’ and ‘Mini-Validation of Repeatability’. For ‘Response Curve’ estimations, our assay experiments were performed in triplicates over seven dilution ranges (Fig. 3). For ‘Mini-validation of Repeatability’ assay experiments were similarly performed in triplicates, at three dilution (concentration) levels – “High”, “Medium”, and “Low”. These were repeated on five different days (Fig. 4). In both ‘Response Curve’ and ‘Mini-validation of Repeatability’, we assessed three transitions for each peptide.

MRMPlus computes performance estimates on two levels for each targeted peptide: 1) each associated transition level and 2) the summation observed transition values. Computed results are presented in tables in which the rows are performance measures for the respectively assayed peptide transition, and the columns are the computed measures of analytical performance. (Additional file 5: Table S5, and Additional file 6: Table S6). It should be noted that no absolute values are reported when the peptide transition values are summed with respect to ‘PVSpec.maxDeviation’, ‘PVSpec.meanAtMaxDev’, ‘PVSpec.dayAtMaxDev’, and ‘PVSpec.concLevelAtMaxDev’ performance measures, because these are specific to transitions.

For ‘Response Curve’ performances, limit of detection across peptide transitions span several magnitudes with minimum LOD reported as 0.000524, and maximum reported as 2293.62 (Additional file 5: Table S5, ‘LOD.value’ column). In all cases, LOD was assessed using spiked-in concentrations (Additional file 5: Table S5, ‘LOD.usedSpiked’ column) as pre-curve blank mass spectrometry runs reported no detectable levels. This is possibly due to the mass spectrometer's sensitivity or resolving power. A lower of limit of quantification (Additional file 5: Table S5, ‘LLOQ.CaliPoint’ column) could not be estimated for peptide transitions YLGNATAIFFLPDEGK (2.b9.1), AGPNGTLFVADAYK (2.y10.1), and INNTHALVSLLQNLNK (3.b6.1). We observed an almost absolutely linear response across all transitions (Additional file 5: Table S5, ‘Curve.Rsquared’ and ‘Curve.SlopeStdErr(%)’ columns) with minimum standard error percent of the slope of the response curve reported as 0.0030 % and maximum as 4.3979 %.

For ‘Mini-Validation of Repeatability’, MRMPlus reports the intra-assay coefficient of variation, inter-assay coefficient of variation, and the total coefficient of variation for each peptide transition at respectively specified low, medium, and high concentration levels. The lowest total CV at the validated lower limit of quantification 3.3918 was found associated with peptide transition YLGNATAIFFLPDEGK (2.y8.1) (Additional file 6: Table S6, ‘ValidatedLLOQ.concLevel’ and ‘totalCV.Me’ columns).

The CPTAC ADWG recommends that for a transition to be deemed specific to a peptide, in all samples above the lower limit of quantification, no transition ratio (ratios of peak areas of different transitions of same peptide) in each sample at all concentrations should deviate >30 % from the mean. MRMPlus reports the maximum deviation percentage observed for each transition; such that a deviation above the specified 30 % indicates non-specificity of such transition to the associated peptide.

Thomas et al. [24] – recently published, provides a complete description of a study of ours demonstrating the utility of MRMPlus.

### Comparing MRMPlus to PanoramaWeb

We compared MRMPlus’ results with comparable outputs derived from PanoramaWeb [25] on our data (Additional file 9: Table S7 and Additional file 10: Table S8). MRMPlus and PanoramaWeb derived exactly the same results for estimation of ‘Repeatability’ performance. With respect to ‘Response Curve’ performance estimation MRMPlus’ and PanoramaWeb results varied. These may particularly be due to their differing methods of estimating linearity. However, Both MRMPlus’ and PanoramaWeb report excellent statistical measure of how close their data are fitted to the regression line – indicated by similar R-squared estimation.

## Conclusions

MRMPlus facilitates MRM assay development. By aligning its workflow with Skyline, a previously established tool for building, analyzing, and refining targeted mass spectrometry methods and the resulting data, MRMPlus streamlines and simplifies assay performance estimation. Future developments of MRMPlus are anticipated to be integrated to the Skyline open-source tool for an even more streamlined analytical workflow.

## Availability and requirements

**Project home:**https://bitbucket.org/paiyetan/mrmplusgui, and https://bitbucket.org/paiyetan/mrmplusgui/downloads

**Operating system:** Platform independent

**Programming Language:** Java

**License:** The BSD 3-Clause License

**Other requirements:** Please see documentation here - https://bitbucket.org/paiyetan/mrmplusgui/wiki/Home.

**Any restrictions to use by non-academics:** None

## Additional files

Additional file 1: Table S1. Sample Skyline-derived response curve experiment input to MRMPlus. (TXT 128 kb) Additional file 2: Table S2. Sample user-defined response experiment metadata file. (TXT 1 kb) Additional file 3: Table S3. Sample user-defined dilution file. (TXT 130 bytes) Additional file 4: Table S4. Sample Skyline-derived repeatability experiment input to MRMPlus. (TXT 199 kb) Additional file 5: Table S5. Sample MRMPlus computed assay performance metrics for the response curve experiments. (TXT 28 kb) Additional file 6: Table S6. Sample MRMPlus computed assay performance metrics for assay repeatability experiments. (TXT 13 kb) Additional file 7: Figure S1. MRMPlus visualizations. (PNG 176 kb) Additional file 8: Figure S2. MRMPlus visualization showing a sample global performance view across all assayed peptide transitions. The x-axis represents individual peptide transitions (including summed transitions). The y-axis represent the computed coefficient of variations (inter-assay, intra-assay, and total) at a median level concentration. (PNG 67 kb) Additional file 9: Table S7. Comparing and contrasting ‘Repeatability’ measures between MRMPlus and PanoramaWeb. (XLSX 9 kb) Additional file 10: Table S8. Comparing and contrasting ‘Response’ measures between MRMPlus and PanoramaWeb. (XLSX 9 kb)
