# Supplementary figures and images for: MRMPlus: an open source quality control and assessment tool for SRM/MRM assay development
Source: BMC Bioinformatics. 2015 Dec 12;16:411. doi: 10.1186/s12859-015-0838-z (PMC4676880; doi:10.1186/s12859-015-0838-z)

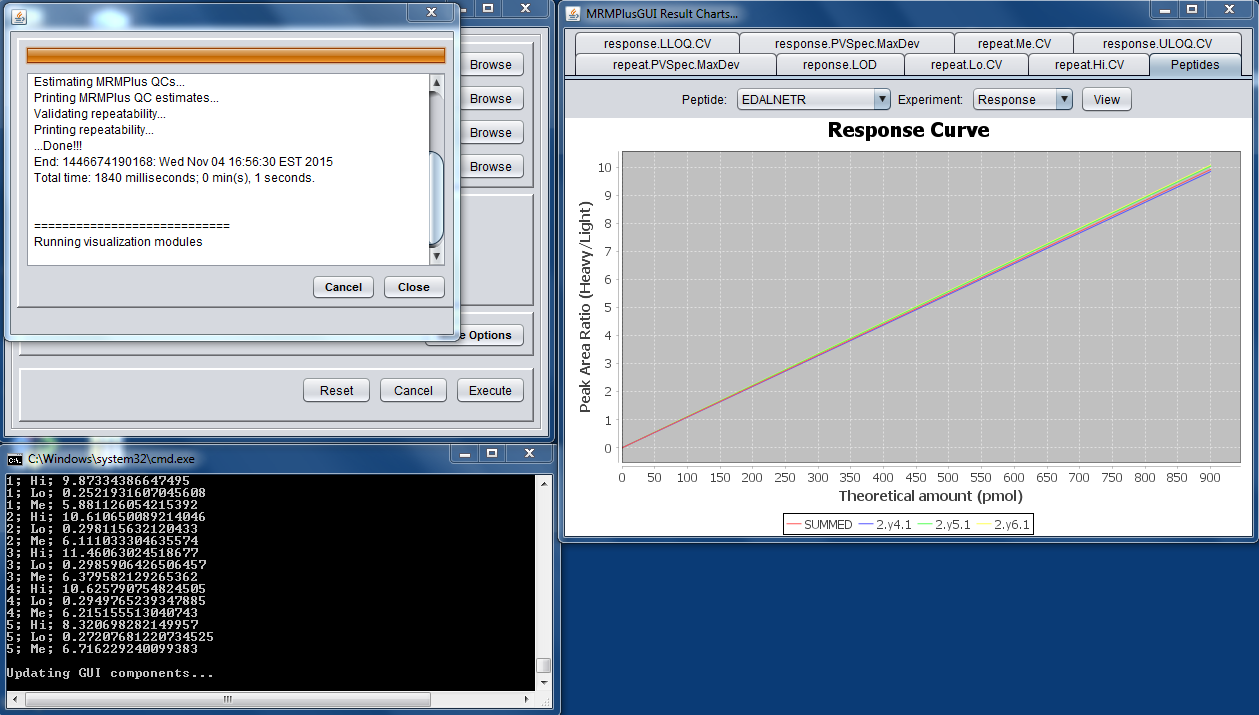

Supplement: Additional file 7: Figure S1. — MRMPlus visualizations. (PNG 176 kb) [file 12859_2015_838_MOESM7_ESM.png]

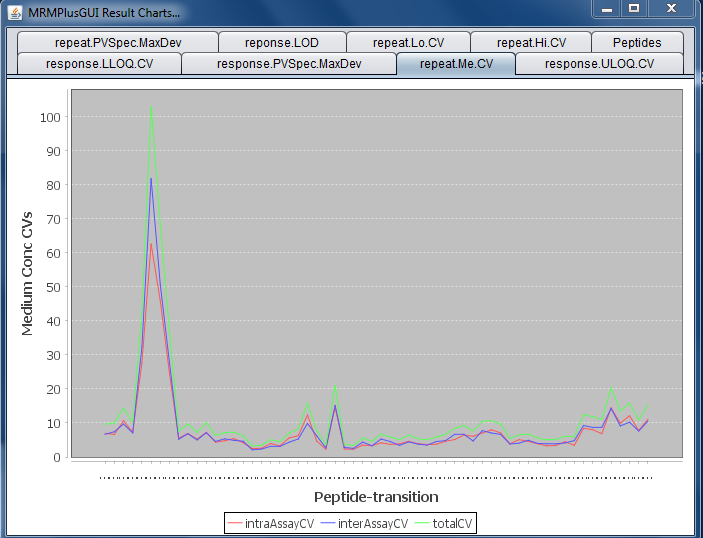

Supplement: Additional file 8: Figure S2. — MRMPlus visualization showing a sample global performance view across all assayed peptide transitions. The x-axis represents individual peptide transitions (including summed transitions). The y-axis represent the computed coefficient of variations (inter-assay, intra-assay, and total) at a median level concentration. (PNG 67 kb) [file 12859_2015_838_MOESM8_ESM.png]
